# Lung health, tobacco, and related products: gaps, challenges, new threats, and suggested research

**DOI:** 10.1152/ajplung.00101.2020

**Published:** 2020-04-01

**Authors:** Simone St Claire, Hebe Gouda, Kerstin Schotte, Ranti Fayokun, Dongbo Fu, Cherian Varghese, Vinayak M. Prasad

**Affiliations:** ^1^Department of Health Promotion, World Health Organization, Geneva, Switzerland; ^2^Department of Non-Communicable Diseases, World Health Organization, Geneva, Switzerland

**Keywords:** electronic cigarettes, heated tobacco products, lung health, tobacco control

The tobacco epidemic is one of the biggest public health challenges the world has ever faced, killing more than 8 million people around the world every year. In 2017, tobacco killed 3.3 million users and people exposed to secondhand smoke from lung-related conditions including chronic respiratory diseases, tracheal, bronchus and lung cancers, respiratory infections and tuberculosis. Despite the strong body of evidence around the harms of tobacco use to lung health from direct and passive exposure to tobacco smoke, there are still several knowledge gaps that need to be bridged through targeted research, for example, tobacco and nicotine addiction, surveillance and monitoring, waterpipe tobacco, heated tobacco products, electronic nicotine delivery systems, inhalation of aerosols, cessation aids, and dual use. Operational and implementation research can help make current efforts and interventions, that we know work, more effective and more efficient. Tobacco use is on the decline, but the rate of reduction is not fast enough and it is apparent that the agreed global target of 30% reduction in tobacco use among adults by 2025 will not be met. Therefore, accelerated research is needed to crystalize the impact of tobacco control towards reducing tobacco use prevalence across populations.

## GLOBAL BURDEN OF TOBACCO USE

The tobacco epidemic is one of the biggest public health challenges the world has ever faced, killing more than 8 million people around the world every year. More than 7 million of those deaths are the result of direct tobacco use while around 1.2 million are the result of nonsmokers being exposed to secondhand smoke (8).

## TOBACCO SMOKE EXPOSURE IMPACTS LUNG HEALTH FOR EVERYONE—NOT JUST SMOKERS

In 2017, tobacco killed 3.3 million users and people exposed to secondhand smoke from lung-related conditions including chronic respiratory diseases, tracheal, bronchus and lung cancers, respiratory infections and tuberculosis (9).

### Lung Cancer

Tobacco smokers are up to 22 times more likely to develop lung cancer in their lifetime, compared with nonsmokers (7, 11, 12, 15, 19, 21). Tobacco smoking is the leading cause of lung cancer, responsible for over 90% of lung cancer deaths in countries where smoking is prevalent in both sexes and claiming 1.2 million lives every year (4, 31). Nonsmokers exposed to secondhand smoke at home or in the workplace have a 30% higher risk of developing lung cancer (18, 27).

### Chronic Obstructive Pulmonary Disease

One in five smokers will develop chronic obstructive pulmonary disease (COPD) in their lifetime (25), and almost half of COPD deaths are attributable to smoking (8). People who started smoking in their youth or were exposed to secondhand smoke during childhood, and had frequent infections of the lower respiratory tract as a result, are especially susceptible to developing COPD (5).

### Tuberculosis

Tobacco smoking more than doubles the risk of developing tuberculosis and increases the risk of recurrent episodes of tuberculosis (TB), as well as the risk of mortality. Exposure to secondhand smoke also increases the risk of TB disease (16, 33).

### Asthma

In people living with asthma, tobacco smoking further restricts activity, contributes to work disability, and increases the risk of severe asthma requiring emergency care. Further, around one in nine asthma deaths can be attributed to tobacco smoking (8).

## THE CHANGING LANDSCAPE OF TOBACCO AND TOBACCO CONTROL

The burden caused by tobacco on people’s health is well understood, but the way tobacco is used and the diversity of tobacco and related products available in many markets are rapidly evolving. Increasingly, waterpipes, commonly known as shisha or hookah, can be found in cafes in cities around the world and are increasingly consumed by females (23). A variety of smokeless tobacco products, which vary in their characteristics relative to conventional smokeless tobacco products, are also available in some markets, including in some European countries. Novel and emerging nicotine, non-nicotine and tobacco products, which are operated using electronic systems, have also been aggressively marketed in many countries around the world. These include:

*1*) Electronic nicotine delivery systems (ENDS) and electronic non-nicotine delivery systems (ENNDS), commonly referred to as e-cigarettes, are devices that heat a liquid to create an aerosol that is inhaled by the user. E-cigarette aerosols typically contain nicotine and toxic substances that are harmful to both users and nonusers who are exposed to the aerosols secondhand (34, 35). People who use these products in combination with smoking conventional cigarettes, which is the most common form of e-cigarette use, are exposed to the toxic chemicals from two or more products (29).

There is growing evidence that ENDS use is associated with lung injuries, with some evidence of acute lung injury, lipoid pneumonia, eosinophilic pneumonia, liquid pneumonia, and bronchiolitis obliterans (2, 10, 17, 24, 26, 28). Furthermore, additional evidence suggests ENDS could have implications for other aspects of lung health (3, 14, 20). E-cigarettes could also be a major threat to lung health, especially in unregulated environments. For example, in the latter half of 2019, the United States investigated an outbreak of lung injuries associated with the use of e-cigarettes or vaping products, which to date has claimed about 70 lives. These injuries, termed EVALI, (e-cigarette or vaping product use associated lung injury) have since been reported to have strong links with vitamin E acetate, with investigations ongoing as to whether there are other chemicals of concern that may have contributed to these injuries.

*2*) Heated tobacco products (HTPs) are tobacco products that produce aerosols containing nicotine and toxic chemicals when tobacco is heated or when a device containing tobacco is activated. These aerosols are inhaled by users during a process of sucking or smoking involving a device. They contain the highly addictive substance nicotine, as well as non-tobacco additives and are often flavored. The tobacco may be in the form of specially designed cigarettes (e.g., “heat sticks” and “neo sticks”) or pods or plugs. Currently, there is no evidence to demonstrate that HTPs are less harmful than conventional tobacco products.

## MORE RESEARCH IS NEEDED TO IMPROVE LUNG HEALTH TO IMPACT TOBACCO CONTROL

Tobacco use is on the decline, but the rate of reduction is not fast enough and it is apparent that the agreed global target of 30% reduction in tobacco use among adults by 2025 will not be met. Therefore, accelerated research is needed to crystalize the impact of tobacco control towards reducing tobacco use prevalence across populations. Operational and implementation research can help make current efforts and interventions, that we know work, more effective and more efficient.

At the clinical level, despite the strong body of evidence around the harms of tobacco use to lung health from direct and passive exposure to tobacco smoke, there are still several knowledge gaps that need to be bridged through targeted research. For example:

- *Tobacco and nicotine addiction*. More research is needed to better understand the many aspects of tobacco and nicotine addiction and how we can help more people to quit tobacco and/or nicotine use in order to reduce their cravings for tobacco and hence exposure to the toxic substances that contribute to the development of lung disease.
- *Tobacco surveillance and monitoring*. Although tobacco surveillance and other monitoring systems are well established, innovations are needed to identify faster and easier methods of assessing tobacco use in the population and to measure quit rates more effectively in an effort to reduce morbidity and mortality attributed to lung-related conditions. These findings will help to advance the development of innovative cessation techniques, reach more people who want to quit, and improve medication and behavioral treatments.
- *Waterpipe*. Contrary to popular belief, the smoke that emerges from a waterpipe contains numerous toxicants known to cause lung cancer, heart disease, and other diseases. Given that there are millions of waterpipe smokers, with waterpipe tobacco use prevalence rates as high as 14% in some countries, and that regular waterpipe use is increasing in several regions across the globe (30), there is surprisingly little research addressing the health impacts of direct and secondhand waterpipe smoking, including on the lungs. Additionally, not much is known about the long-term effects of intermittent or infrequent waterpipe use or the cumulative harm of dual use of waterpipe tobacco and cigarettes (30). Therefore, further research to better understand the health effects associated with smoker exposure and exposure to secondhand waterpipe smoke, risks of transmission of communicable diseases, and the effectiveness of specific prevention and cessation strategies is imperative.
- *HTPs*. HTP aerosols contain the same substances found in conventional cigarette smoke, as well as other chemicals not found in conventional cigarette smoke, which may have associated health effects. Independent assessment of industry data shows that more than 20 harmful and potentially harmful chemicals are significantly higher than in reference cigarette smoke. Additionally, these products are highly variable and some of the toxicants found in the emissions of these products may have implications for lung health. Therefore, further research is needed to understand the short-term and long-term effects of direct and passive exposure to the aerosols of these products on lungs and the respiratory system.
- *ENDS*. ENDS on their own are associated with increased risk of cardiovascular diseases (1), lung disorders (32) and adverse effects on the development of the fetus during pregnancy (35). However, robust research is needed to quantify risk and provide a clear answer on the health effects associated with long-term exposure to ENDS’ toxic emissions.
- *Inhalation of aerosols*. Another area of research that could be prioritized is investigation into the inhalation of the aerosols of ENDS and HTPs. This is important because even though most of the flavors used in these products have been tested for ingestion and generally recognized as safe, their effects when inhaled are not known. Further, the particles of e-cigarette emissions have been reported to be very small, which could have implications for the depth of penetration into the lungs. Therefore, research into e-cigarette emission particle size and the depth of penetration into the lungs will help to better understand the health effects of these small particles.
- *Cessation aids*. In recent years, products such as HTPs, ENDS, and ENNDS have been promoted as “reduced harm” products and/or products that can help people quit conventional tobacco smoking. While HTPs are themselves tobacco products, switching from conventional tobacco products to HTPs does not constitute cessation. As for ENDS/ENNDS, at the time of writing, the evidence on the use of these products as potential cessation aids is inconclusive. ENDS/ENNDS should not be promoted as a cessation aid until adequate evidence is available and the public health community can agree on the effectiveness of those specific products. Independent and well-designed studies are needed to assess ENDS/ENNDS as cessation aids.
- *Dual use*. The link between smoking and lung cancer, as well as other respiratory diseases, is well established. However, even though the concurrent use of e-cigarettes with conventional cigarettes, which is the most prevalent form of dual use (13), has been reported to expose such users to a combination of the toxic chemicals in e-cigarettes and conventional tobacco smoke (6, 22), the effects of this exposure have not been well studied. Therefore, further research is needed to establish the impact of such exposures on the lungs, given that it is often postulated that dual and/or poly users are more susceptible to developing similar diseases to those linked with exposure to tobacco smoke.

## WHAT CLINICIANS CAN DO TO SUPPORT TOBACCO CONTROL EFFORTS AND PREVENT THE HARMS DUE TO TOBACCO

Health professionals are uniquely positioned to enhance prevention and cessation strategies, and they are encouraged to at least assess tobacco use status, advise on the effects of tobacco use and exposure to secondhand smoke, and assist in tobacco cessation. Much of the required infrastructure for promoting tobacco cessation measures, such as a primary healthcare system, exists in most countries (36). Integrating brief advice into existing systems is one of the first feasible and affordable actions countries can take to strengthen comprehensive national tobacco cessation systems (37).

In addition, as trusted opinion leaders in their communities, health professionals can also act as tobacco-free role models for the public and advocate for tobacco control and ensure that measures such as smoke-free environments in clinics, hospitals, and other public places are respected.

## WORLD HEALTH ORGANIZATION’S RESPONSE TO THE TOBACCO EPIDEMIC

The World Health Organization (WHO) Framework Convention on Tobacco Control (WHO FCTC) provides a strong, concerted response to the global tobacco epidemic and its enormous health, social, environmental and economic costs. To help countries implement the WHO FCTC, WHO introduced the MPOWER technical package. Examples of key strategies in this approach include banning tobacco advertising, promotion, and sponsorship and significantly increasing taxes on tobacco products. Since the MPOWER measures were introduced more than a decade ago, the number of people covered by at least one of these effective tobacco control measures has more than quadrupled, from 1 billion in 2007 to 5 billion in 2018 (37). Over the past two decades, global tobacco use has fallen from 1.397 billion in 2000 to 1.337 billion in 2018, or approximately 60 million people. For the first time, the number of male tobacco users, which had previously been increasing every year, is on the decline (38).

Despite significant global progress, many countries are still not adequately implementing policies that can save lives from tobacco, and progress in meeting the global target set by governments to reduce the prevalence of tobacco use by 30% by 2025 remains off track. Currently, only 32 countries are on track to reach the 30% reduction target (38). The lag in global progress is due in part to tobacco industry’s long history of systematic, aggressive, sustained, and well-resourced opposition to tobacco control measures (39), including efforts to subvert life-saving tobacco control measures. It does this by deploying a wide variety of tactics to obstruct, delay, weaken, or undermine political commitments and tobacco control measures taken by countries at international, regional, national, and subnational levels. Commitment to countering industry interference is fundamental to successful implementation of effective tobacco control measures in accordance with the WHO FCTC. In the clinical context, requiring disclosure of, and clearly communicating, funding sources for research institutions, academics, and scientific studies to prevent unseen biases in science on which policy may be based is essential to counter tobacco industry interference. Tobacco control efforts need to be strengthened globally, and in some countries accelerated, to reduce tobacco-attributable morbidity and mortality. By taking action to implement effective measure to reduce tobacco use, governments, health professionals and civil society can and will save millions of lives each year.

## DISCLAIMERS

The authors are staff members of the World Health Organization. The authors alone are responsible for the views expressed in this article and they do not necessarily represent the decisions, policy or views of the World Health Organization.

## DISCLOSURES

No conflicts of interest, financial or otherwise, are declared by the authors.

## AUTHOR CONTRIBUTIONS

S.S.C. drafted manuscript; S.S.C., H.G., K.S., R.F., D.F., C.V., and V.M.P. edited and revised manuscript; V.M.P. approved final version of manuscript.
